# A mixed-utility theory of vote choice regret

**DOI:** 10.1007/s11127-018-0571-z

**Published:** 2018-06-16

**Authors:** Damien Bol, André Blais, Jean-François Laslier

**Affiliations:** 1 Department of Political Economy, King’s College London (KCL), 30 Aldwych (Bush House), London, WC2B 4BG UK; 2 Départment de Science Politique, Université de Montréal (UdeM), C.P. 6128, Succursale Centre-Ville, Montreal, QC H3C 3J7 Canada; 3 Paris School of Economics, 48 Boulevard Jourdan, 75014 Paris, France

**Keywords:** Voting behaviour, Expressive voting, Vote choice regret, D72, D84, D91

## Abstract

The paper builds upon an original pre- and post-election survey that we conducted before and after the 2015 Canadian election. Directly after Election Day, we asked Canadians for which party they voted, and whether they regret their choice. We find that 39% of them are not perfectly happy with their decision, and 4% even say that they made a bad decision. We show that the propensity to regret can be explained by a mixed-utility theory, whereby voters attempt to maximize a mixture of instrumental and expressive utilities. Our study contributes to the literatures on voting behaviour and political economy, which usually considers that voters are either instrumental or expressive, but not both at the same time.

**Electronic supplementary material:**

The online version of this article (10.1007/s11127-018-0571-z) contains supplementary material, which is available to authorized users.

## Introduction

Voters sometimes regret their choices. That issue has made the headlines in the United Kingdom after the Brexit referendum on membership in the European Union (*The Independent* 2016). Numerous voters said that they voted “Leave” but would have voted differently if they had known that the Leave option was going to win. Indeed, most pre-referendum polls predicted a (close) victory for the “Remain” camp. That situation echoes the 2002 presidential election in France, during which the far-right candidate Jean-Marie Le Pen advanced to the second round of voting to the surprise of most journalists and political pundits. Again, many left-wing voters expressed regret for not having voted for the center-left candidate Lionel Jospin, who failed to qualify for the second round (Radio France International 2002). The recent election of Donald Trump as the President of the United States also has its share of vote choice regrets, usually among Trump voters who thought Clinton would win (Vox 2017).

Yet, vote choice regret^1^ is not specific to extraordinary elections with surprising outcomes. As we show in this paper, many voters regret their choices in ‘normal’ elections too. In an original survey conducted the week directly following the 2015 Canadian election, we find that 39% of voters express some regret about their choices; 4% of them even say that they made a bad decision. At that moment, voters knew which party would form the government, but the cabinet had not yet been assembled, and no policy had been implemented. In other words, the only relevant event that happened between vote choices and the post-election survey was the public release of the electoral result.^2^

A situation in which a large proportion of voters regret their choices the week after the election certainly is not desirable. Vote choice regret cannot be triggered by the actions of the new government, as the government had not yet been formed; it must come from the election itself, and likely means that the voters wrongly anticipated the electoral results.^3^ Moreover, the situation is particularly problematic when it appears that the electoral outcome would have been different if regretting voters had a chance to change their votes.^4^ For example, the evidence suggests that the Remain option would have won if another Brexit referendum had been organized a few weeks after the actual referendum. Data from the British Election Study (2016) show that around 6% of Leave voters were ready to change their vote. In this paper, we do not claim to evaluate whether the outcome of the 2015 Canadian election would have been different if voters who expressed regret in our survey could have changed their votes. However, we offer an explanation for why some voters express regret. This is, to our knowledge, the first study that provides an explanation of vote choice regret.

The point we want to make is that we can explain the presence of regret for rational individuals with stable preferences if we assume that voters seek to maximize a mixed-utility function composed of both instrumental and expressive motives (on the definition of ‘instrumental’ and ‘expressive’, see Hamlin and Jennings 2011; Hillman 2010; and below). By mixed-utility, we mean that voters gain some benefit from voting instrumentally, plus some benefits from voting expressively. Using their single vote, they cast ballots to maximize their overall benefit, which is a mixture of instrumental and expressive benefits. In other words, (at least some) voters who regret their choice do so because they realize *ex post,* and in view of the results, that they did not vote for the party that maximizes their mixed utility. The conflict between the two motives explains why some people end up voting for a party that does not maximize their utility, and subsequently can regret their choice upon learning the election results. In order to prove our point, we construct a simple and parsimonious mixed-utility theory of vote choice that relies on district-level results and answers to only one survey question (i.e., party liking). We show that the theory is supported by the data: it explains a substantial part of vote choice regret even when we control for other relevant variables.

This paper thus provides new evidence about an old, yet timely, debate about voters’ motivations. Classic economic theories, in particular the popular Hotelling-Downs model of spatial competition (Downs 1957; Hotelling 1929), assume that voters have instrumental motives, in the sense that they care about the outcome of the election. In the seminal work of Duverger (1954), such instrumental motives explain why some voters strategically desert their preferred party in plurality elections. When their preferred party is too small and has few chances of winning, they cast a vote for their preferred large party as to maximize their chances of influencing the election’s outcome.^5^

Attempts have been made to add expressiveness as another source of motivation to voting models (Brennan and Hamlin 1998; Piketty 2000; Laslier and Weibull 2013; Schuessler 2001). Following that literature, the expressive voter does not consider how other voters will vote (contrary to the instrumental voter who wants to affect the outcome but can do so only if her vote is decisive), as what matters to her is the preference conveyed by her individual vote regardless of outcome. The empirical literature that relies on survey data finds that some voters cast vote as if they were purely instrumental, and others as if they were purely expressive (Drinkwater and Jennings 2007; Kan and Yang 2001). Experimental studies also make the distinction between instrumental and expressive voters, showing that ‘pivotality’ affects the relative proportion of both types (Copeland and Laband 2002; Feddersen et al. 2009; Fischer 1996; Tyran 2004). Other studies find evidence that some voters do vote expressively, in utilizing ‘natural’ quasi-experiments, such as the ban of a party in Spain (Arenas 2016), or the qualification-threshold for the second round in France (Pons and Tricaud 2017). The literature on strategic voting also sometimes considers that voters derive some utility from voting expressively, instead of strategically, which in turn explains the frequency of situations of incomplete coordination (Eggers and Vyvian 2018; Myatt 2007).

So, ways to establish the relevance of expressive voting empirically are possible. However, it is impossible to know whether observations of votes are the result of the coexistence of two types of ‘pure voters’ or of one type of ‘mixed-utility voters’, who are motivated by both instrumental and expressive benefits, and end up voting in one way or another depending on the circumstances. The study of vote choice regret offers a unique opportunity in that respect. Unlike vote choice, which yields only one data point about voters’ preferences, vote choice regret gives a more fine-grained measure of whether the voter is ambivalent regarding several possible options.

This paper is organized as follows. First, we present our mixed-utility theory, which aims to explain vote choice regret. Second, we describe the data that we use to test the theory. Third, we show that the empirical evidence strongly supports our argument. Finally, we conclude in deriving the contributions of this study for the literatures on political economy and voting behaviour.

## A mixed-utility theory of vote choice regret

Our theory supposes that a voter derives utility from two sources: how much she likes the party for which she votes (expressive utility), and how much her vote makes a difference to the party that is elected (instrumental utility).^6^ These utilities are defined below. To illustrate the difference between the two, let us just take the example of two fictitious voters: André is an expressive voter and votes for party A because A is the party that defends the policies he stands for; Jean-François is an instrumental voter and he votes for party B because B is the party that will implement his most preferred policies. The key difference between the two is that André is not affected by the probability of winning of the different parties when he decides for which party to vote; he derives satisfaction from the act of voting for his preferred party. By contrast, Jean-François does consider the different parties’ probabilities of winning, as he wants his vote to make influence which party is elected. That goal typically implies voting for a party that has some chance of winning in his local district.

If voters are partly expressive and partly instrumental, they might face a two-choice alternative: voting for their instrumental (like Jean-François) or expressive choice (like André). If we go back to our examples, let us assume that Jean-François is partly instrumental and partly expressive. He favors party A. Unfortunately for him, party A is so small that it has virtually no chance of winning. Therefore, he decides to vote for party B because he thinks that party B has some chance of winning. He will regret his choice if he realizes, after the election, that party B had no greater chance of winning than party A. Similarly, André, who is also partly instrumental and partly expressive, will regret his choice if he realizes, after the election, that his vote could have elected party B. Just like Jean-François he also thinks that he would be better off under the policies of party B than under the policies of the party that won the election.

Let us now define the utilities formally. We consider that the utility for voter *i* to vote for party *a* is:

1 $$U_{ia} = \phi U^{expr}_{ia} + \, (1 - \phi ) \, U^{inst}_{ia}$$

The parameter *ϕ* can possibly vary between 0 and 1; it is the relative weight the voter gives to expressive utility (compared to instrumental utility). A rational voter votes for the party that maximizes the function. When *ϕ *= 1, she is a purely expressive voter: she will vote for the party she likes the most. When *ϕ *= 0, she is a purely instrumental voter: she will vote for the party she likes more among the top two parties running in the district. In a plurality system with single member districts like Canada, the voter can make a difference only for one of the two parties that we label as ‘viable’. By logical implication, a third party is always further away from victory than one of the top two. Therefore, if the voter wants to influence the outcome of the election, she needs to vote for one of the viable parties.^7^ When *ϕ* is somewhere between 0 and 1, the voter sometimes votes for the party she likes the most and sometimes votes for the party she likes the most among viable parties. Which choice she makes depends on the values of her instrumental and expressive utilities (see below).

But when does she regret her vote choice? A voter regrets her choice if she realizes, after the election, that she made a choice that did not maximize her overall utility. In other words, a voter *i* regrets voting for party *a* if the utility of voting for some other party *ω* is higher than the utility of voting for *a*. Party *ω* can be either the party she likes the most or the party she likes the most among the viable parties. It is because *i* did not realize at the time of voting that she did not make the right choice. Furthermore, *i* does not regret voting for *a* if it was the party with the highest utility (*a *= *ω*). It is when her expected utility *ex ante*, which led her to vote for party *a*, does not coincide with the realized electoral outcome that she regrets her vote, typically when she over- or underestimates the margin of victory between the top two candidates in the district.

Our mixed-utility theory then predicts that voters regret their choice in two situations:

(Situation 1): She votes for the party she likes the most among the two viable parties in her district, though this party is not her favorite party. Then she realizes, after the election, that her vote did not help the party for which she voted to be elected, because the margin of victory between her district’s two viable parties is large.
(Situation 2): A voter votes for the party she likes the most, though that party is not among the two viable parties in her district. Then she realizes, after the election, that her vote could have made a difference between the two viable parties, because the margin of victory between them is small.^8^

In both situations, regret occurs because of the tradeoff the voter has to make between her two motives. At first glance, we could think that the voter might be purely instrumental in Situation 1 and purely expressive in Situation 2. But that would be wrong. In Situation 1, the voter regrets voting for the party she likes the most among the viable parties because her vote did not help to elect that party. By saying that, we assume that she would have been better off voting for the party she liked the most among all competing parties. But that is true only if we consider that she also has expressive motives; otherwise, she would not even bother thinking about voting for her preferred party. Similarly, in Situation 2, she regrets voting for her preferred party because her vote could have made a difference between the two viable parties. That is possible only if we consider that she also has an instrumental motive.

It is important to note that we are making two assumptions here: (1) the voter’s preferences for parties do not change between her vote and the moment at which she experiences regret, and (2) the voter knows the identities of the two viable parties in her district. Those two assumptions are necessary, as their negation would lead to other regret predictions. For example, a voter who voted for the party she likes the most but realizes, after the election, that she does not like it anymore would regret her vote even if she is purely expressive. Also, a voter who votes for what she thinks is a viable party but realizes, after the election, that it is not viable would also regret her vote even if she is purely instrumental.

In the empirical part of this paper, we relax the foregoing assumptions, by adding control variables that seek to capture other sources of regret. First, we add a variable capturing whether respondents report having ambivalent preferences for different parties before the election. We expect that ambivalent voters are more likely to change preferences after the election. Also, we add a variable capturing whether respondents are able to identify correctly the viable parties in their districts before the election.^9^ We observe that more than 80% of the respondents are able to correctly identify the two parties with the greatest chances of winning. However, we do not assume that the voter is perfectly able to predict the electoral results. In fact, our predictions rely on the idea that the voter is able to correctly identify which parties are viable in her district, even if she wrongly anticipates the vote margin between the top two parties.

Now that we have identified the situations in which a voter with a mixed utility regrets her choice, we need to define the expressive and instrumental utilities represented in Eq. 1. As mentioned above, those utilities rely on a few measures: district-level electoral results and answers to one survey question: how much do you like each of the competing parties on a scale from 0 (really dislike) to 10 (really like)? That question was asked in the pre-election survey conducted during the week preceding the election. We denote by *L*_*ia*_ how much voter *i* likes party *a.* The intuition is straightforward: the voter derives a positive instrumental benefit when her vote makes a difference as to which party is elected in her district, and a positive expressive benefit when she votes for the party she likes the most. Also, it is important to note that since we are interested in the probability that the voter regrets her choice after the election, the electoral results are known.

The voter derives expressive utility when she likes the party for which she votes. We consider that the more she likes the party the greater is her expressive utility. However, not everybody has the same point of reference when they answer a survey question. Some people might like most parties, some people might dislike them all. We thus need to compare the way the voter likes party *a* to a reference point. For that purpose, we use how much the voter likes all parties other than party *a* (and we denote that average by $\overline{L}_{i - a}$).

2 $$U^{expr}_{ia} = \, L_{ia} {-}\overline{L}_{i - a}$$

The voter derives instrumental utility from her vote when she is pivotal (i.e., when the vote difference between the top two parties in her district is zero or one). That is the only situation in which her vote changes the election’s outcome. In real-life elections, when the number of voters is large, a single voter virtually is never pivotal. However, previous research shows that voters tend to overestimate the pivotality of their votes (Blais 2000; Castanheira 2003; Duffy and Tavits 2008; Kan and Yang 2001). Like them, we consider that such overestimation is related to the closeness of the election. When the two viable parties are very close, the probability of being pivotal approaches 1, and that probability declines further as the difference in vote shares between the top two parties increases. Formally, we measure the closeness of the race between the top two parties by:

3 $$C_{1,2} = \, \left( {1 \, {-} \, \left( {V_{1} {-} \, V_{2} } \right)} \right)^{2}$$

where *V*_1_ is the vote share of party 1, the leading party in the district, and *V*_2_ is the vote share of party 2, the second-leading party in the district. Thus, *C*_1,2_ takes the value 1 when the two parties receive the same number of votes (the election is very close), and 0 when party 2 had 0% of the votes (the election is not close at all). We square the term to account for convexity of the closeness function: if the gap is two percentage points, then closeness is nearly as great as when the gap is 1 percentage point; if the gap is 10 percentage points, the election is much less close than a gap of 9% points.^10^

When *a* is one of the viable parties, the voter can think that her vote can make a difference, especially if the election is close. In that situation, instrumental utility is the difference between how much *i* likes *a* (denoted *L*_*ia*_) and how much she likes the other viable party (denoted *L*_*i* (1 *or* 2_)) multiplied by the closeness of the election (*C*_12_).^11^ If *a* is not one of the viable parties, voting for it cannot make a difference, so the voter’s instrumental utility is nill.

4 $$\begin{array}{*{20}l} {{\text{For}}\;a = 1, \, 2:\quad U^{insrt}_{ia} = \, C_{12} \left( {L_{ia} - \, L_{i \, (1 \, or \, 2)} } \right)} \hfill \\ {{\text{For}}\;a \ne 1, \, 2:\quad U^{instr}_{ia} = \, 0} \hfill \\ \end{array}$$

Now that we have defined the expressive and instrumental utilities, we can write the voter’s overall utility that she derives from voting for party *a*. Recall that $\phi$ denotes the relative weight given by the voter to the expressive utility.

5 $$\begin{aligned} & {\text{For}}\;a = 1, \, 2:\quad U_{ia} = \, \phi \, \left( {L_{ia} - \overline{L}_{i - a} } \right) + \, \left( {1 - \, \phi } \right) \, C_{12} \left( {L_{ia} - \, L_{i \, (1 \, or \, 2)} } \right) \\ & {\text{For}}\;a \ne 1, \, 2:\quad U_{ia} = \, \phi \, \left( {L_{ia} - \overline{L}_{i - a} } \right) \\ \end{aligned}$$

We compute this overall utility for all competing parties, so as to find out the utility associated with the party that is the voter’s best option (party *ω*) and then compare that to the utility associated with the party for which she ends up voting (party *a*). The problem with this approach is that for each voter, the identity of party *ω* may vary depending on the value of the parameter *ϕ*. As mentioned above, it can be either the party that the voter likes the most among the two viable parties (*ω *= 1, 2) or the party that she likes the most among all parties. More precisely, for each voter, a threshold *t*_*i*_ exists such that:^12^

6 $$\begin{aligned} &{\text{If}}\;\phi /\left( {1 - \, \phi } \right) \, < \, t_{i} ,\;\omega \;{\text{is}}\;{\text{the}}\;{\text{party}}\;{\text{that}}\;i\;{\text{likes}}\;{\text{the}}\;{\text{most}}\;{\text{among}}\;1\;{\text{and}}\;2. \hfill \\ & {\text{If}}\;\phi /\left( {1 - \, \phi } \right) \, > \, t_{i} ,\;\omega \;{\text{is}}\;{\text{the}}\;{\text{party}}\;{\text{that}}\;i\;{\text{likes}}\;{\text{the}}\;{\text{most}}\;{\text{among}}\;{\text{all}}\;{\text{parties}}. \hfill \\ \end{aligned}$$

The relations in (6) have an important implication for the empirical part of the paper. Since *ω* depends on the value of *ϕ*, we need to compute, for each possible value of *ϕ* (from 0 to 1), and for each voter, the overall utility associated with each party. Once that is done, we can replace, for each value of *ϕ,* the utility associated with party *ω* and compare it to the utility associated with party *a.* The larger the difference between the utility of voting for *a* and the utility of voting for *ω* the more likely the voter is to regret her choice.

Finally, note that the instrumental and expressive utilities vary between − 10 and + 10. They thus can be negative in principle. Utilities are negative when the voter votes for her least preferred of the two viable parties (instrumental utility) or for a party that she likes much less than other parties on average (expressive utility). Since we weight each term by *ϕ* and *1*–*ϕ*, overall utility also can vary from − 10 to + 10. A negative utility is very rare in the data but, as a robustness test, we reproduce the analyses by dropping the respondents who voted for a party that gives them a negative utility overall (less than 5% of the 3058 respondents).

## The data

To test our mixed-utility theory, we use data from an original pre- and post-election panel survey conducted during Canada’s 2015 national election. The survey was conducted within the Making Electoral Democracy Work project (Blais 2010). Canadian federal elections are held under plurality rule in single-member districts, just like in the United States, the United Kingdom or India. In Canada, multiple parties compete in national elections. It is thus logically harder for voters to cast utility-maximizing votes.

The Liberal Party and its leader Justin Trudeau defeated the incumbent Prime Minister Stephen Harper of the Conservative Party in the 2015 Canadian election. The Liberal Party received 39% of the votes and 54% of the seats; it thus was able to form a majority government. The Conservative party received 32% of the votes and 29% of the seats and formed the official opposition. The New Democratic Party (center-left) came in third; it obtained 24% of the votes and 19% of the seats. The Bloc Québécois (Quebec independentist party), which competes only in Quebec, came in fourth with 5% of the votes and 3% of the seats. Finally, the Green Party obtained 3% of the votes and one seat (in British Columbia). No other party received more than 1% of the votes at the national level.

After having ruled the country for almost 10 years, the incumbent Prime Minister was particularly unpopular in the Canadian electorate. For a long time, the polls predicted that the opposition party, the New Democratic Party, would win the election. However, about a month before Election Day, the Liberal Party gained considerable support and appeared to have a strong chance of winning the election, though it was unclear whether it would receive enough seats to form a majority government.

In our survey, we recruited respondents in the three largest provinces of the country (Ontario, Quebec and British Columbia) using pre-existing online panels.^13^ We adopted recruitment quotas based on age, education, gender and region to ensure the socio-demographic representativeness of the sample. We conducted the first wave of the survey during the two weeks preceding Election Day, and the second wave the week after. In each province, about 1800 respondents completed the pre-election questionnaire, and among them about 1400 completed the post-election questionnaire.

As to measure vote choice regret, we used the responses to one survey question. In the post-election questionnaire, we asked respondents for which party they had voted. Then, right after that question, we asked them whether they thought they made a good decision in voting the way they did. Four response categories to that question were offered: the vote choice was a “very good”, a “fairly good”, a “fairly bad”, or a “very bad” decision.

Table 1 reports the aggregated responses to the regret question. It reveals that most respondents (61%) seem perfectly happy with the decision and say that they made a very good decision. However, we find that 36% of them acknowledge that they made only a fairly good decision, 3% a fairly bad decision, and 1% a very bad decision. Therefore 39% of voters are not completely happy with their choices. That proportion is similar to what Blais and Kilibarda (2016) find in other countries using the same question. They find that the proportion of voters expressing some regret ranges from 31 to 54%, depending on the country and the election.

**Table 1:** Regret in the 2015 Canadian national election

For which party did you vote? […] Do you think your choice decision was	Proportion (%)	N
Very good	61	1867
Fairly good	36	1089
Fairly bad	3	86
Very bad	1	16

In this paper, we use three different measures of regret based on the answers to the post-election survey question described above. In the main analysis, we use the original response categories of the variable (recoded from 0 “very good” to 3 “very bad”). Then, in a series of supplementary tests, we use two other versions of the variable. First, we dichotomize the responses in distinguishing between respondents who say that they made a bad decision (fairly bad or very bad, 4%) and those who say that they made a good decision (fairly good or very good, 96%). We label that dummy variable ‘big regret’. Second, we dichotomize the responses in distinguishing respondents that are not completely happy with their votes (those who say that they made a fairly good decision, a fairly bad decision, or a very bad decision, 39%) and those who are not (those who indicated having made a very good decision, 61%). We label that dummy variable ‘small regret’. Expressing some doubt about the wisdom of one’s own decision, even by saying that it was ‘a fairly good decision’, is already a sign of regret.

To operationalize our mixed-utility theory, we use the answer to only one survey question. In the pre-election questionnaire, we asked respondents how much they liked each of the main competing parties on a scale from 0 to 10 (from “really dislike” to “really like”).^14^ The main parties in Canada are the Liberal Party, the Conservative Party, the New Democratic Party, and the Green Party. A fifth party competed only in Quebec: the Bloc Québécois.

The details of all of the questions, including the distribution of responses, are reported in the online appendix. We also need the score (vote share) of each party in the respondent’s district to compute the closeness of the election between the top two parties. We identify the district based on the postal code of the respondent.

## Empirical analysis

We test the theory presented above against our survey data from Canada. To do so, we adopt an approach similar to a joint estimation of the parameters of interest. However, instead of using a built-in command, we decompose the different steps to be more transparent. First, we report the main analysis using the original four-category dependent variable; second, we report a series of supplementary tests showing that the results are robust to a wide range of specifications and other operationalizations of the dependent variable.

### Main analysis

In line with our theory, we estimate the following regression model using an OLS specification:

$${\text{Degree}}\;{\text{of}}\;{\text{Regret}}_{\text{i}} = \beta_{0} + \beta_{1} \left( {U_{i\omega } - \, U_{ia} } \right) \, + \beta_{k} \;{\text{Controls}}_{i} \; + \;\varepsilon$$

*U*_*ia*_ is the utility the respondent receives in voting for party *a* (the party for which she votes) and *U*_*iω*_ is the utility the respondent might have received in voting for her best vote choice (party *ω*). For the sake of clarity, we label the term *U*_*iω*_–*U*_*ia*_ as the ‘utility difference between vote and optimal party’. For each value of *ϕ* (for the definition of ϕ, see above), some respondents vote for their best vote choice; the term thus equals 0 for them. However, some respondents vote for a party that is not their best vote choice; they have a positive utility difference, which means that they do not vote for the party that maximizes their utility. The higher the value of the term, the larger is the difference between vote cast and optimal party. Therefore, we expect *β*_*1*_ to be positive.

We also include control variables to account for potential confounding effects. First, we add two control variables to capture other sources of regret. We add a variable that is a difference between how much the respondent likes her most preferred party compared to how much she likes her second most preferred party (‘ambivalence’). The rationale is that if a respondent does not have a clear party preference, that is, she is ambivalent about two or more parties, the more likely she is to change her preference ordering. Hence, her expressed regret might be explained by the change in party preference and not to our mixed-utility theory. The findings confirm that indeed those who are ambivalent and therefore are more likely to change their preferences are more inclined to regret their choice after the election.

Second, we include a variable capturing whether the respondent has accurate expectations regarding the viability of her preferred party. In the pre-election survey, we asked respondents to evaluate the chances of winning for all competing parties (the question wording is in the online appendix). We then check their expectations in light of the actual electoral results. The variable ‘correct expectations’ takes the value of 1 if the respondent accurately identifies her preferred party as one of two parties with the highest chances of winning or not, and 0 in all other situations. We expect that respondents who have erroneous expectations were more likely to regret their choices. We also control for the overall satisfaction of respondents with the parties competing in the election. We expect that respondents who do not like any party are more likely to regret their choices. We create a dummy variable that takes the value of 1 when the respondent does not rate any party at 5 or more on the 0–10 liking scale. The findings also confirm that voters with correct expectations regarding electoral results are less likely to regret their choices while those who dislike all of the parties are more prone to express regret.

Furthermore, we also add Canadian provincial dummies to account for the nested character of our data, as well as several sociodemographic control variables: the respondent’s age, gender, and whether she has a university degree (all asked in the pre-election survey questionnaire). We also include dummy variables capturing the party for which she votes. We expect voters to experience less regret when the party for which they voted won the election and will form the government. It is also important to note that some of these variables have a statistically significant effect on the probability of a respondent regretting her vote choice: age (negative), dissatisfaction with parties in general (positive), ambivalence (positive), and the act of voting for either the Liberal Party (negative) or the New Democratic Party (positive). As mentioned above, the Liberal Party won the 2015 Canadian federal election and the New Democratic Party lost its status of official opposition. The evidence suggests that voters derive some positive ‘social’ benefits from voting for the national winner and negative ‘social’ benefits from voting for the national loser, which is in line with studies of bandwagon effects (Bartels 1985; McAllister and Studlar 1991).

Table 2 reports the regression results. As mentioned above, which party is identified as *ω* depends on how heavily the respondent weights expressive utility relative to instrumental utility. Therefore, we have different results for each value of *ϕ*. In Table 2, we present the estimates of the regression model specified above with *ϕ* varying between 0 and 1 (with steps of 0.1) When *ϕ *= 1, the respondent is purely expressive; when *ϕ *= 0, the respondent is purely instrumental.

**Table 2:** Regression results for each value of *ϕ*

	*ϕ *= 0	*ϕ *= 0.1	*ϕ *= 0.2	*ϕ *= 0.3	*ϕ *= 0.4	*ϕ *= 0.5	*ϕ *= 0.6	*ϕ *= 0.7	*ϕ *= 0.8	*ϕ *= 0.9	*ϕ *= 1
Utility difference between vote and optimal party	0.027**	0.035**	0.043**	0.053**	0.063**	0.070**	0.072**	0.070**	0.067**	0.064**	0.059**
	(0.006)	(0.007)	(0.007)	(0.008)	(0.008)	(0.009)	(0.009)	(0.008)	(0.008)	(0.007)	(0.007)
Age	− 0.002**	− 0.002**	− 0.002**	− 0.002**	− 0.002*	− 0.002*	− 0.002*	− 0.002*	− 0.002*	− 0.002*	− 0.002*
	(0.001)	(0.001)	(0.001)	(0.001)	(0.001)	(0.001)	(0.001)	(0.001)	(0.001)	(0.001)	(0.001)
Gender	0.008	0.008	0.008	0.009	0.009	0.009	0.009	0.009	0.008	0.008	0.007
	(0.020)	(0.020)	(0.020)	(0.020)	(0.020)	(0.020)	(0.020)	(0.020)	(0.020)	(0.020)	(0.020)
University degree	− 0.015	− 0.015	− 0.014	− 0.014	− 0.013	− 0.013	− 0.014	− 0.015	− 0.016	− 0.017	− 0.018
	(0.020)	(0.020)	(0.020)	(0.020)	(0.020)	(0.020)	(0.020)	(0.020)	(0.020)	(0.020)	(0.020)
Dissatisfaction with parties	0.280**	0.281**	0.281**	0.280**	0.279**	0.276**	0.274**	0.272**	0.272**	0.273**	0.274**
	(0.040)	(0.040)	(0.040)	(0.040)	(0.040)	(0.039)	(0.039)	(0.039)	(0.039)	(0.039)	(0.039)
Ambivalence	0.057**	0.057**	0.057**	0.057**	0.058**	0.059**	0.060**	0.060**	0.060**	0.060**	0.060**
	(0.005)	(0.005)	(0.005)	(0.005)	(0.005)	(0.005)	(0.005)	(0.005)	(0.005)	(0.005)	(0.005)
Correct expectations regarding viability	− 0.047	− 0.047	− 0.049	− 0.053*	− 0.057*	− 0.061*	− 0.064*	− 0.066**	− 0.065**	− 0.062*	− 0.059*
	(0.026)	(0.026)	(0.026)	(0.025)	(0.025)	(0.025)	(0.025)	(0.025)	(0.025)	(0.025)	(0.025)
Party choice (Conservative Party as reference)											
NDP	0.102**	0.100**	0.098**	0.096**	0.094**	0.092**	0.092**	0.092**	0.091**	0.091**	0.091**
	(0.029)	(0.029)	(0.029)	(0.029)	(0.029)	(0.029)	(0.029)	(0.029)	(0.029)	(0.029)	(0.029)
Liberal Party	− 0.169**	− 0.171**	− 0.173**	− 0.177**	− 0.181**	− 0.186**	− 0.189**	− 0.192**	− 0.195**	− 0.198**	− 0.200**
	(0.026)	(0.026)	(0.026)	(0.026)	(0.026)	(0.026)	(0.026)	(0.026)	(0.026)	(0.026)	(0.026)
Bloc Québécois	− 0.045	− 0.045	− 0.044	− 0.042	− 0.040	− 0.038	− 0.036	− 0.035	− 0.034	− 0.034	− 0.034
	(0.047)	(0.046)	(0.046)	(0.046)	(0.046)	(0.046)	(0.046)	(0.046)	(0.046)	(0.046)	(0.046)
Green Party	0.024	0.022	0.021	0.020	0.020	0.023	0.027	0.030	0.032	0.033	0.034
	(0.051)	(0.051)	(0.051)	(0.050)	(0.050)	(0.050)	(0.050)	(0.050)	(0.050)	(0.050)	(0.050)
Province dummies	YES	YES	YES	YES	YES	YES	YES	YES	YES	YES	YES
Constant	0.669**	0.665**	0.663**	0.662**	0.664**	0.667**	0.672**	0.676**	0.677**	0.676**	0.676**
	(0.060)	(0.060)	(0.060)	(0.059)	(0.059)	(0.059)	(0.059)	(0.059)	(0.059)	(0.059)	(0.059)
R^2^	0.118	0.120	0.122	0.125	0.128	0.131	0.133	0.133	0.133	0.133	0.133
N	3058	3058	3058	3058	3058	3058	3058	3058	3058	3058	3058

Entries are coefficient estimates from OLS regressions. The dependent variable is the degree of regret about vote choice. Standard errors are in parentheses. *p < 0.05; **p < 0.01

Table 2 shows that, at each value of *ϕ*, the coefficient associated with the utility difference between vote and optimal party is positive and statistically significant at a level of *p *< 0.01. However, some coefficients are larger than others, indicating that the effect is stronger for some values of *ϕ* than for others. Typically, the coefficients are larger for non-extreme values. The coefficient is particularly small when we assume that voters are mostly instrumental (*ϕ* approaching 0). It also is true, to a lesser extent, when we assume that voters are mostly expressive (*ϕ* approaching 1). In fact, the model that has the largest coefficient is the one where we suppose that the respondents weight expressive utility at 60% and instrumental utility at 40%. We perform various SUEST tests to evaluate which coefficients are statistically different from the largest one (at *ϕ *= 0.6) and find that that applies to all regressions assuming that respondents are 60% or more instrumental (*ϕ *= 0.4) or 90% or more expressive (*ϕ *= 0.9). In other words, the best models are those assuming voters maximize a mixed-utility function. Moreover, all supplementary tests below show that the best model always is the one in which mixed-utility is assumed.

It is important to note that results of Table 2 do not mean that everybody in our sample weights expressive utility at 60% (and instrumental utility at 40%) in their vote choices. It means that the average weighting is 60% expressive, but it is possible that few respondents operate with this precise value of *ϕ *= 0.6. However, our data do not allow us to estimate parameter *ϕ* for each respondent individually. However, in the subsample analyses that we report below, we find that the optimal value of *ϕ* is always between 0.6 and 0.8. Those results are consistent with the view that a substantial proportion of voters have a mixed-utility composed of both expressive and instrumental elements. Even in sub-groups for which we could have expected that expressive motives prevail (for example, respondents with little political knowledge), the value of *ϕ *= 0.6 fits the data best. We also perform below several robustness tests in which we change some regression parameters, such as the way closeness is calculated and find that the optimal value of *ϕ* always is around 0.6 (see below).

We now address the question how much regret our theory is able to predict. In theory, the main variable, the utility difference between actual vote and optimal vote, can vary between 0 (when party *a* = *ω*) and 20. That extreme positive value is almost impossible to reach. It is only reached when the expressive and instrumental utilities of party *ω* are maximal (expressive utility = 10 and instrumental utility = 10), but the respondent ends up voting for a party she strongly dislikes (expressive utility = − 10), and which is her lesser liked party among the two viable parties (instrumental utility = − 10). In reality, however, the utility difference between actual and optimal votes varies between 0 and 14, with 82% of the respondents having a value of 0 (meaning that they vote for the party that maximizes their mixed-utility),^15^ with a mean of 0.35 and a standard deviation of 1.14. It is interesting to note that 27% of utility-maximizing voters really like their favorite party (party liking of 10). For them, there is not much of a choice. However, 29% of voters in that group do not have a strong preference: there is no party that they like more than 7.

**Fig. 1:**
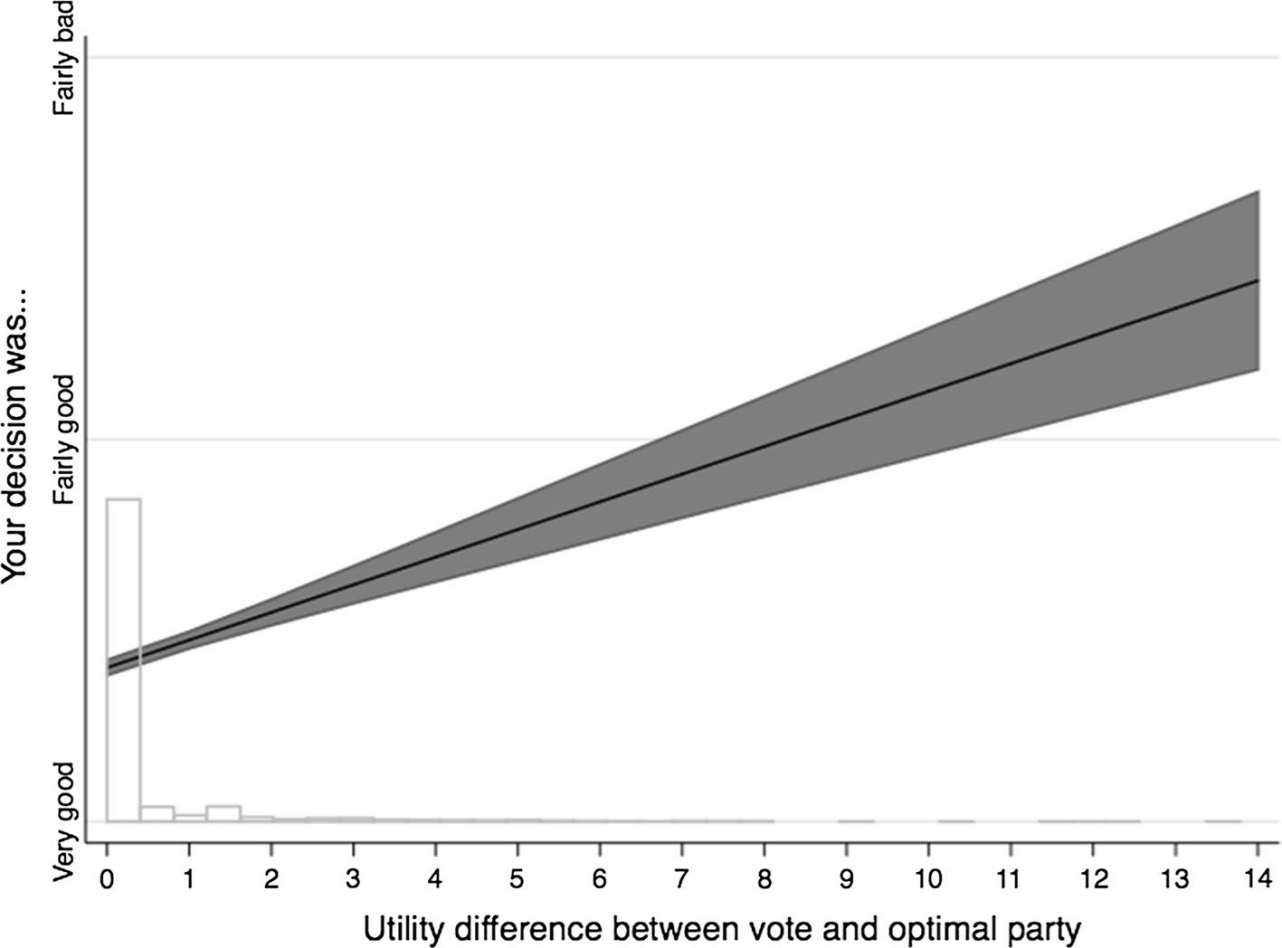
Predicted probabilities of small regret (*ϕ *= 0.6). *Note*: The line is the predicted values of an OLS regression and the shaded area is the 95% confidence interval. Other independent variables are set at their mean. The histogram at the bottom shows the distribution of the utility difference between vote cast and optimal party

In Fig. 1, we report the estimates of the benchmark model, which assumes that respondents weight their expressive utility at 60% and their instrumental utility at 40%. It shows that the predicted value increases radically between the two empirical extremes (from 0.4 to 1.4). This is the equivalent of moving from a situation in which the respondent is likely to say that she made either a very good or a fairly good decision to another in which she is likely to say that she made a fairly good or a fairly bad decision (first-difference test, *p *< 0.01).

Second, as mentioned above, 82% of the survey respondents maximized their mixed utility if we assume that *ϕ *= 0.6. For the remaining respondents, the mean is 1.97 and the standard deviation is 2.02. A shift of two standard deviations around that mean produces an increase in the predicted value of the dependent variable from 0.4 to 0.7. That also is a substantial increase as it means moving from a situation wherein the respondent is more likely to say that she made a good decision to another one in which she is more likely to say that she only made a fairly good decision (first-difference test, *p *< 0.01).

### Supplementary tests

We conduct a series of supplementary tests to ensure that the results presented above are robust. In the online appendix, we show the coefficient associated to the utility difference between vote and optimal party for each value of *ϕ* (A4 and A6), and the full results of the regressions for which this is the highest (A5 and A7).

First, we re-estimate the regression of the main analysis with alternative dependent variables. We re-estimate an OLS regression with (1) the ‘big regret’ dummy variable (fairly or very bad decision versus fairly or very good decision), and (2) the ‘small regret’ dummy variable (fairly bad, very bad, fairly good versus very good decision). Then, we estimate the following models predicting the degree of regret as in the main analysis: (1) defining the closeness of the race between the top two parties in the district with a linear function instead of concave function and (2) excluding respondents who voted for a party with a negative utility. Table A5 shows that the coefficient for the utility difference between vote and optimal party is similar in the five alternative regressions described above. It varies from 0.02 (big regret) to 0.14 (no negative utility) and always is statistically significant at *p *< 0.01 level.

Second, we re-estimate the OLS regression of the main analysis for various subsamples. First, we distinguish respondents by their level of campaign knowledge. To do so, we use a survey question of the post-election questionnaire that asks respondents to match three actual campaign slogans with one of the three main parties (the Liberal Party, the Conservative Party and the New Democratic Party). We distinguish between respondents who are able to correctly match all three slogans (high knowledge), and those who are not able to do so (low knowledge). We expect the effect of the utility difference between vote and optimal party to be larger for knowledgeable voters, because our theory assumes that voters are aware of the electoral results the week following the election. Knowledgeable voters are more likely to closely follow the election than others. Also, they are more likely to have accurate expectations regarding the results of the upcoming election and thus are more likely to cast votes that maximize their utility.

Then, we also distinguish between respondents who are in a district where the vote margin between the second and third party is larger than 0.1, and those who are not. We expect our mixed-utility theory to work best when a clear margin emerges between the second and third party because we define viability by considering only the two top parties in the district. Table A7 shows that the coefficient for the utility difference between vote and optimal party always is positive and statistically significant at a level of *p *< 0.01. We find that *ϕ* never takes the value of 1 (purely expressive) nor 0 (purely instrumental). It varies between 0.6 and 0.7. This supplies additional evidence that a substantial proportion of voters have mixed utility functions comprising both expressive and instrumental elements.

## Conclusion

The topic of vote choice regret has made the headlines in the weeks following important elections, such as the Brexit referendum or the election of Donald Trump as the President of the United States. A substantial number of voters have said that they would have voted differently if they had known the electoral outcome. Such a situation is obviously problematic, especially when the election is so close that the regretful voters were numerous enough to change the electoral result.

This paper is the first to examine systematically the sources of vote choice regret. To do so, we develop a new theory, which supposes that voters have mixed-utility functions composed of both instrumental and expressive motives. We then test this theory using unique pre- and post-election survey data from the 2015 Canadian national election. In the post-election wave, conducted in the immediate aftermath of the election, we asked respondents whether they regret their vote choices or not. We find that 39% of them were not perfectly happy with their decision, and 4% even said that they made a bad decision. The 2015 Canadian national election is different from the emblematic elections with surprising outcomes mentioned above in the senses that the result was not unexpected and that more than two credible options were available. We show that our mixed-utility theory explains an important fraction of the regret expressed after the election results were known. We believe that, with some minor amendments, our theory can apply to all elections, including those held under proportional representation rules. Various studies find that some voters also seek to influence the electoral outcome in those systems (Bargsted and Kedar 2009; Duch et al. 2010; Indridason 2011). However, the story would need to be more complex given that coalition agreements also have the capacity to influence the electoral outcome.

Our paper also contributes to the literature on voting behavior. Traditionally, this literature assumes, sometimes implicitly, that individual voters have either instrumental motives, in the sense that they care about the outcome of the election, or expressive motives, in the sense that they care about voting for a party or a candidate they like. However, very few studies integrate the two in a single model (for an exception, see Myatt 2007).

Finally, our paper shows that including a regret question in surveys can help to understand what motivates people to vote in the first place. We believe our approach has great potential for behavioral studies in general. Many theoretical and experimental studies investigate human decisions and behaviors by making assumptions about why people act the way they do. Asking people whether they regret a decision immediately after the decision was taken in real life can be a good way of learning about their motives. The regret concept also is used in game theory. For example, a Nash equilibrium is a situation in which nobody regrets her decision. Therefore, we believe that more surveys should include a regret question, thereby allowing researchers to test their underlying assumptions regarding people’s motives.

## Electronic supplementary material

Below is the link to the electronic supplementary material.

Supplementary material 1 (DOCX 2766 kb)

## Footnotes

1 This paper is about the regret of voting for a given party; we call this vote choice regret. Our mixed-utility theory relates directly to the ‘regret theory’ proposed by Loomes and Sugden (1982) in the sense that we use a similar definition of regret in the presence of uncertainty. However, unlike them, we do not assume that voters anticipate the possibility of regretting their votes before voting. Closer to our topic, a small literature studies regret in the context of voter turnout. That literature usually argues that the decision to turn out is explained mostly by intrinsic individual motives, such as interest in politics and a sense of civic duty (Blais 2000; Kamm and Schram 2019; Riker and Ordeshook 1968).

2 For this paper, we rely on Canadian data. In Canada, elections are held under single-member district plurality rule. This electoral system has the advantage of creating straightforward incentives for voters: they vote in a district, the party with most votes in that district wins the seat, and the party with most seats across districts forms the government. We believe that the argument we develop also applies to elections held under multi-member district proportional representation. However, the story would need to be more complex given that more than two viable parties compete per district, and voters need to consider potential governing coalitions to make their vote choice decisions (Bargsted and Kedar 2009; Duch et al. 2010; Indridason 2011). It is important to note that Canada has no history of governing coalitions at the national level. In the 2015 election analysed in this paper, it was clear no coalition would be necessary even if the Liberal Party would not have won a majority of seats.

3 In theory, a voter could also regret her choice because she changes her preference after having voted. In this study, we look at vote choice regret under the assumption that voters’ preferences remain the same during the week following the election. In the empirical analysis, we relax that assumption by adding control variables capturing other sources of regret, including a change in preferences.

4 Another reason to study vote choice regret is that it can undermine voters’ satisfaction with democracy. In our post-election survey, we asked respondents whether they are satisfied with the way democracy works in their country on a scale from 0 (not satisfied at all) to 10 (very satisfied). In the online appendix (A3), we report a negative association between regret and satisfaction with democracy. On average, satisfaction with democracy declines as voters become more regretful (*p *< 0.01). However, the association is small because the predicted value of satisfaction moves from 7 (vote choice is a very good decision) to 5.5 (vote choice was a very bad decision). This evidence shows that vote choice regret is a different attitude than satisfaction with democracy, although the two are associated.

5 In reality, an individual voter has virtually no chance of affecting the outcome of an election. In this paper, we adopt an approach similar to other studies that approximate the probability of being pivotal by the closeness of the election (Blais 2000; Castanheira 2003; Kan and Yang 2001; and below).

6 Our theory is focused on the utility associated with the voter’s own vote. The utility derived from seeing a candidate winning is not specified directly.

7 This line of reasoning is similar to the one adopted by studies of strategic voting (Cox 1997). One limitation is that it neglects districts in which electoral competition involves three parties. If the third party is strong enough, the voter might still make a difference in the electoral outcome by voting for the third party. In a test of robustness, we extend the analyses by separating the respondents into two groups depending on the vote margin between the second and third party.

8 We make an extra assumption for this second prediction: the voter is not indifferent between the two viable parties. Given how we compute utilities, indifferent voters have no instrumental utility and therefore cannot experience regret (see below).

9 In the pre-election wave of our survey, we asked respondents what the chances were of winning of each competing party on a scale from 0 (very unlikely) to 10 (very likely). The exact wording of all questions used in our analyses can be found in the online appendix (A1).

10 In a test of robustness, we reproduce the analyses using a linear version of the closeness variable, that is, without squaring the term.

11 In classic models of spatial competition, a voter’s utility usually is assumed to be a function of how her ideal point is from those of the parties or candidates on a potentially *N*-dimensional ideological policy space (Merrill and Grofman 1999). But the most direct indicator of the utility that is associated with a party is how much the voter likes that party. The more a voter likes a party the higher is her utility (Blais et al. 2017).

12 The value of this threshold is found as follows: let *L*_*i*_**** be the best evaluation of all parties and let *L*_*i*_*** be the best evaluation of all viable parties. Let *n* be the number of parties. If *L*_*i*_*** > L*_*i*_*** then, *t*_*i*_* = (*1*–*1*/n) C*_12_*|L*_*i1*_*–L*_*i*2_*|/(L*_*i*_***–L*_*i*_**)* and if *L*_*i*_*** = L*_*i*_***, then *t*_*i*_* =+∞*.

13 Note that if we sum up the vote choices of all survey respondents, we observe that the vote share of the main parties is very similar to the actual electoral results. The largest difference concerns the Conservative Party in Ontario, which is underestimated by four percentage points, and the New Democratic Party in British Columbia, which is overestimated by the same margin. Most discrepancies between reported and actual vote shares are smaller than two percentage points. Those differences supply evidence that the survey sample is not biased in favor or against any particular party. The full distribution of the responses to the vote choice question is provided in the online appendix (A2).

14 By main party, we mean the parties that received at least 1% of the votes in the province. In our analyses, the party-liking variables are rescaled to vary between 0 and 1.

15 It is interesting to note that the proportion is similar to the proportion of ‘correct votes’ that Lau and Redlawsk (1997) find in the United States in their cross-sectional study.

## References

[CR1] Arenas A (2016). Sticky votes. Journal of Economic Behavior & Organization.

[CR2] Bargsted M, Kedar O (2009). Coalition-targeted Duvergerian voting: How expectations affect voter choice under proportional representation. American Journal of Political Science.

[CR3] Bartels LM (1985). Expectations and preferences in presidential nominating campaigns. American Political Science Review.

[CR4] Blais A (2000). To vote or not to vote? The merits and limits of rational choice.

[CR5] Blais A (2010). Making electoral democracy work. Electoral Studies.

[CR6] Blais A, Guntermann E, Bodet MA (2017). Linking party preferences and the composition of government: A new standard for evaluating the performance of electoral democracy. Political Science Research and Methods.

[CR7] Blais A, Kilibarda A (2016). Correct voting and post-election regret. PS: Political Science and Politics.

[CR8] Brennan G, Hamlin A (1998). Expressive voting and electoral equilibrium. Public Choice.

[CR9] British Election Study. (2016). *Brexit Britain: British Election Study insights from the post*-*EU referendum wave of the BES internet panel*. Available at: http://www.britishelectionstudy.com/bes-resources/brexit-britain-british-election-study-insights-from-the-post-eu-referendum-wave-of-the-bes-internet-panel/#.WFF-56LJwcb. Last Accessed on 14 December, 2016.

[CR10] Castanheira M (2003). Victory margins and the paradox of voting. European Journal of Political Economy.

[CR11] Copeland C, Laband DN (2002). Expressiveness and voting. Public Choice.

[CR12] Cox GW (1997). Making votes count: Strategic coordination in the worlds’ electoral systems.

[CR14] Downs A (1957). An economic theory of democracy.

[CR15] Drinkwater S, Jennings C (2007). Who are the expressive voters?. Public Choice.

[CR16] Duch RM, May J, Armstrong D (2010). Coalition-directed voting in multi-party democracies. American Political Science Review.

[CR17] Duffy J, Tavits M (2008). Beliefs and voting decisions: A test of the pivotal voter model. American Journal of Political Science.

[CR18] Duverger M (1954). Political parties: Their organization and activity in the modern state.

[CR19] Eggers, A. C., & Vyvian N. (2018). *Who votes more strategically?* Working Paper.

[CR20] Feddersen T, Gailmard S, Sandroni A (2009). Moral bias in large elections: Theory and experimental evidence. American Political Science Review.

[CR21] Fischer AJ (1996). A further experimental study of expressive voting. Public Choice.

[CR22] Hamlin A, Jennings C (2011). Expressive political behaviour: foundations, scope and implications. British Journal of Political Science.

[CR23] Hillman AL (2010). Expressive behavior in economics and politics. European Journal of Political Economy.

[CR24] Hotelling H (1929). Stability in competition. Economic Journal.

[CR25] Indridason IH (2011). Proportional representation, majoritarian legislatures, and coalitional voting. American Journal of Political Science.

[CR26] Kamm A, Schram A, Congleton R, Grofman B, Voigt S (2019). Experimental public choice: Elections. Oxford handbook of public choice.

[CR27] Kan J, Yang CC (2001). On expressive voting: Evidence from the 1988 U.S. presidential election. Public Choice.

[CR28] Laslier J-F, Weibull JW (2013). An incentive-compatible condorcet jury theorem. The Scandinavian Journal of Economics.

[CR29] Lau RR, Redlawsk DP (1997). Voting correctly. American Political Science Review.

[CR30] Loomes G, Sugden R (1982). Regret theory: An alternative theory of rational choice under uncertainty. The Economic Journal.

[CR31] McAllister I, Studlar DT (1991). Bandwagon, underdog, or projection? Opinion polls and electoral choice in Britain, 1979–1987. The Journal of Politics.

[CR32] Merrill S, Grofman B (1999). A unified theory of voting.

[CR33] Myatt DP (2007). On the theory of strategic voting. Review of Economic Studies.

[CR34] Piketty T (2000). Voting as communicating. Review of Economic Studies.

[CR35] Pons, V., & Tricaud, C. (2017). *Expressive voting and its cost: Evidence from runoffs with two or three candidates*. Harvard Business School Working Paper 17–107.

[CR37] Radio France International. (2002). *21 avril*-*5 mai: deux semaines de bouleversements*. Available at: http://www1.rfi.fr/actufr/articles/029/article_14443.asp. Last Accessed on December 14, 2016.

[CR38] Riker WH, Ordeshook PC (1968). A theory of the calculus of voting. American Political Science Review.

[CR39] Schuessler A (2001). The logic of expressive choice.

[CR40] The Independent. (2016). *Brexit: More than one million people want to change their vote from Leave to Remain*. Available at: http://www.independent.co.uk/news/uk/politics/brexit-eu-referendum-bregret-leave-petition-second-remain-latest-will-we-leave-a7105116.html. Last Accessed on December 14, 2016.

[CR41] Tyran J-R (2004). Voting when money and morals conflict: An experimental test of expressive voting. Journal of Public Economics.

[CR42] Vox. (2017). *I voted for Donald Trump, and I already regret it*. Available at: http://www.vox.com/first-person/2017/1/18/14300952/donald-trump-vote-regret. Last Accessed February 21, 2017.

