# Supplementary material for: A mixed-utility theory of vote choice regret
Source: Public Choice. 2018 Jun 16;176(3):461–78. doi: 10.1007/s11127-018-0571-z (PMC6424201; doi:10.1007/s11127-018-0571-z)
Supplement: Supplementary file 1 — Supplementary material 1 (DOCX 2766 kb) [file 11127_2018_571_MOESM1_ESM.docx]

**A mixed-utility theory of vote choice regret**

**Online appendix**

Damien Bol (King’s College London)

André Blais (Université de Montréal)

Jean-François Laslier (Paris School of Economics)

**A1. Survey questions**

**A2. Description of variables**

**A3. Association between regret and satisfaction with democracy**

**A4. Best *ϕ* in supplementary tests: other specifications**

**A5. Supplementary regressions: other specifications**

**A6. Best *ϕ* in supplementary tests: sub-samples**

**A7. Supplementary regressions: sub-samples**

**A1. Survey questions**

Party-liking

On a scale from 0 (don't like at all) to 10 (like a lot), how much do you like the:

- Conservative Party

- New Democratic Party

- Liberal Party

- Green Party

- Bloc Québécois (only in Quebec)

Vote choice

Which party's candidate did you vote for?

- Conservative Party

- New Democratic Party

- Liberal Party

- Green Party

- Bloc Québécois (only in Quebec)

Regret

Given the outcome of the election, do you think that your decision was

- A very bad decision

- A fairly bad decision

- A fairly good decision

- A very good decision

**A2. Description of variables**

|  | **Ontario**  **(N = 1,072)** | **British Columbia**  **(N = 999)** | **Quebec**  **(N = 987)** |
| --- | --- | --- | --- |
| **Party liking (mean/standard deviation)** |  |  |  |
| Conservative Party | 4.00  (3.66) | 3.75  (3.62) | 3.76  (3.40) |
| New Democratic Party | 5.44  (2.79) | 5.20  (3.20) | 5.79  (2.67) |
| Liberal Party | 5.70  (3.11) | 5.68  (2.90) | 5.23  (3.06) |
| Green Party | 5.08  (2.83) | 5.45  (2.75) | 4.30  (2.87) |
| Bloc Québécois | . | . | 4.32  (3.57) |
|  |  |  |  |
| **Vote Choice (%)** |  |  |  |
| Conservative Party | 31 | 29 | 18 |
| New Democratic Party | 20 | 30 | 24 |
| Liberal Party | 45 | 34 | 34 |
| Green Party | 4 | 7 | 3 |
| Bloc Québécois | . | . | 21 |
|  |  |  |  |
| **Regret (%)** |  |  |  |
| A very bad decision | 1 | 1 | 1 |
| A fairly bad decision | 3 | 3 | 3 |
| A fairly good decision | 33 | 36 | 38 |
| A very good decision | 63 | 60 | 59 |

**A3. Association between regret and satisfaction with democracy**

Note: Estimated with an OLS regression predicting satisfaction with democracy (0-10 scale) by the answer to the regret question. The bar is the predicted values of satisfaction with democracy and the shaded area is the 95% confidence interval. N=3,016. The coefficient is -0.41 and is statistically significant at a level *p*<.01.

**A4. Best *ϕ* in supplementary analysis: other specifications**

**
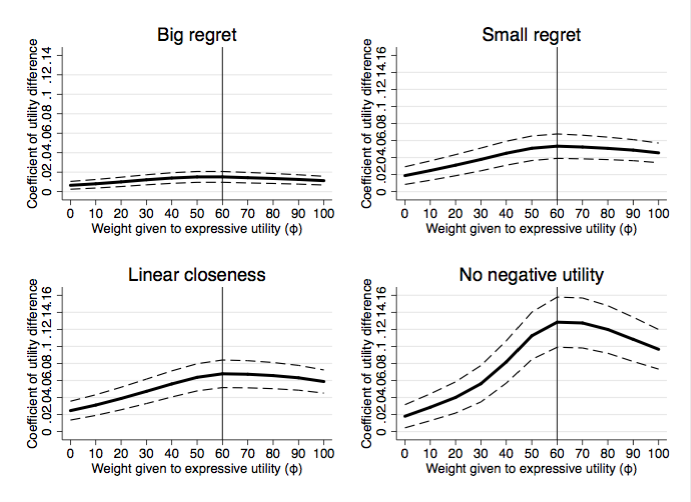
**

Note: The solid line is the coefficient of the utility difference between vote and optimal party of the regression for each value of ϕ in an OLS regression. The dashed lines are the 95%-confidence interval. The horizontal line is the model with the highest coefficient.

**A5. Supplementary regressions: other specifications**

|  | Big regret | Ordinal logit | Linear closeness | No negative utility |
| --- | --- | --- | --- | --- |
|  | *ϕ=0.6* | *ϕ=0.6* | *ϕ=0.7* | *ϕ=0.6* |
|  |  |  |  |  |
| Utility difference | 0.015** | 0.053** | 0.068** | 0.135** |
|  | (0.003) | (0.007) | (0.008) | (0.016) |
| Age | 0.000 | -0.002** | -0.002* | -0.002* |
|  | (0.000) | (0.001) | (0.001) | (0.001) |
| Gender | -0.012 | 0.024 | 0.009 | 0.009 |
|  | (0.006) | (0.017) | (0.020) | (0.020) |
| University degree | -0.016* | 0.006 | -0.014 | -0.012 |
|  | (0.007) | (0.017) | (0.020) | (0.020) |
| Dissatisfaction with parties | 0.047** | 0.213** | 0.274** | 0.275** |
|  | (0.013) | (0.033) | (0.039) | (0.041) |
| Ambivalence | 0.004* | 0.056** | 0.060** | 0.059** |
|  | (0.002) | (0.004) | (0.005) | (0.005) |
| Correct expectations regarding viability | -0.021** | -0.039 | -0.065* | -0.050 |
|  | (0.008) | (0.021) | (0.025) | (0.026) |
| Party choice |  |  |  |  |
| NDP | 0.028** | 0.059* | 0.092** | 0.078** |
|  | (0.009) | (0.024) | (0.029) | (0.029) |
| Liberal Party | -0.024** | -0.162** | -0.189** | -0.197** |
|  | (0.009) | (0.022) | (0.026) | (0.026) |
| Bloc Québécois | 0.000 | -0.037 | -0.036 | -0.031 |
|  | (0.015) | (0.039) | (0.046) | (0.046) |
| Green Party | 0.029 | -0.026 | 0.026 | 0.019 |
|  | (0.016) | (0.042) | (0.050) | (0.051) |
| Province dummies | YES | YES | YES | YES |
| Constant | 0.056** | 0.612** | 0.672** | 0.655** |
|  | (0.019) | (0.050) | (0.059) | (0.060) |
|  |  |  |  |  |
| R^2^ | 0.037 | 0.133 | 0.132 | 0.138 |
| Observations | 3,058 | 3,058 | 3,058 | 2,921 |

Note: Entries are coefficient estimates from OLS regressions. Standard errors are in parentheses. * *p*<0.05, ** *p*<0.01.

**A6. Best *ϕ* in supplementary analysis: sub-samples**

| **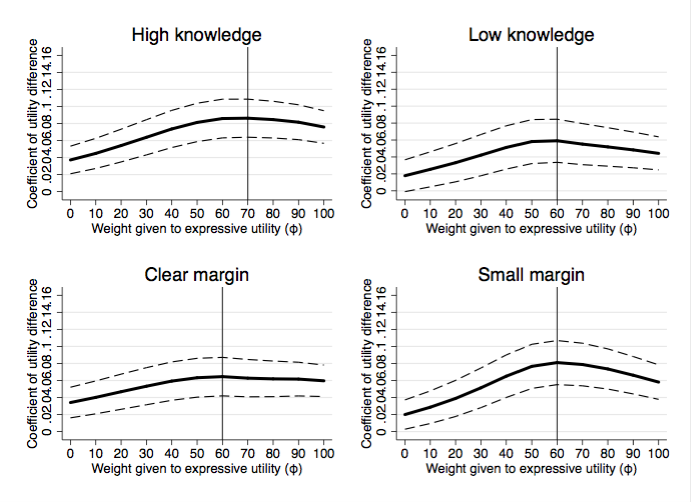** |
| --- |

Note: The solid line is the coefficient of the utility difference between vote and optimal party of the regression for each value of ϕ in an OLS regression. The dashed lines are the 95%-confidence interval. The horizontal line is the model with the highest coefficient.

**A7. Supplementary regressions: sub-samples**

|  | Low knowledge | Low knowledge | Clear margin | No clear margin |
| --- | --- | --- | --- | --- |
|  | *ϕ=0.7* | *ϕ=0.6* | *ϕ=0.6* | *ϕ=0.6* |
|  |  |  |  |  |
| Utility difference | 0.086** | 0.059** | 0.065** | 0.081** |
|  | (0.011) | (0.013) | (0.012) | (0.013) |
| Age | -0.001 | -0.001 | -0.003** | -0.000 |
|  | (0.001) | (0.001) | (0.001) | (0.001) |
| Gender | 0.033 | -0.022 | 0.010 | 0.010 |
|  | (0.027) | (0.030) | (0.027) | (0.028) |
| University degree | -0.003 | -0.013 | -0.017 | -0.010 |
|  | (0.026) | (0.031) | (0.028) | (0.029) |
| Dissatisfaction with parties | 0.314** | 0.238** | 0.296** | 0.252** |
|  | (0.063) | (0.052) | (0.055) | (0.057) |
| Ambivalence | 0.061** | 0.058** | 0.058** | 0.062** |
|  | (0.006) | (0.008) | (0.007) | (0.007) |
| Correct expectations regarding viability | -0.083* | -0.053 | -0.059 | -0.068* |
|  | (0.036) | (0.036) | (0.040) | (0.033) |
| Party choice |  |  |  |  |
| NDP | 0.036 | 0.134** | 0.080 | 0.114** |
|  | (0.039) | (0.043) | (0.042) | (0.041) |
| Liberal Party | -0.192** | -0.194** | -0.238** | -0.125** |
|  | (0.034) | (0.041) | (0.035) | (0.040) |
| Bloc Québécois | -0.002 | -0.043 | -0.063 | 0.000 |
|  | (0.077) | (0.061) | (0.132) | (0.053) |
| Green Party | -0.106 | 0.151* | -0.001 | 0.056 |
|  | (0.068) | (0.074) | (0.072) | (0.071) |
| Province dummies | YES | YES | YES | YES |
| Constant | 0.638** | 0.709** | 0.763** | 0.567** |
|  | (0.080) | (0.087) | (0.083) | (0.084) |
|  |  |  |  |  |
| R^2^ | 0.137 | 0.128 | 0.151 | 0.119 |
| Observations | 1,590 | 1,468 | 1,534 | 1,524 |

Note: Entries are coefficient estimates from logit regressions. Standard errors are in parentheses. * *p*<0.05, ** *p*<0.01.
